# E2F mediates enhanced alternative polyadenylation in proliferation

**DOI:** 10.1186/gb-2012-13-7-r59

**Published:** 2012-07-02

**Authors:** Ran Elkon, Jarno Drost, Gijs van Haaften, Mathias Jenal, Mariette Schrier, Joachim AF Oude Vrielink, Reuven Agami

**Affiliations:** 1 Division of Gene Regulation, The Netherlands Cancer Institute, Plesmanlaan 121, Amsterdam, 1066 CX, The Netherlands; 2 The Centre for Biomedical Genetics, UMCU, Stratenum 3.217, Universiteitsweg 100, 3584 CG Utrecht, The Netherlands

## Abstract

**Background:**

The majority of mammalian genes contain multiple poly(A) sites in their 3' UTRs. Alternative cleavage and polyadenylation are emerging as an important layer of gene regulation as they generate transcript isoforms that differ in their 3' UTRs, thereby modulating genes' response to 3' UTR-mediated regulation. Enhanced cleavage at 3' UTR proximal poly(A) sites resulting in global 3' UTR shortening was recently linked to proliferation and cancer. However, mechanisms that regulate this enhanced alternative polyadenylation are unknown.

**Results:**

Here, we explored, on a transcriptome-wide scale, alternative polyadenylation events associated with cellular proliferation and neoplastic transformation. We applied a deep-sequencing technique for identification and quantification of poly(A) sites to two human cellular models, each examined under proliferative, arrested and transformed states. In both cell systems we observed global 3' UTR shortening associated with proliferation, a link that was markedly stronger than the association with transformation. Furthermore, we found that proliferation is also associated with enhanced cleavage at intronic poly(A) sites. Last, we found that the expression level of the set of genes that encode for 3'-end processing proteins is globally elevated in proliferation, and that E2F transcription factors contribute to this regulation.

**Conclusions:**

Our results comprehensively identify alternative polyadenylation events associated with cellular proliferation and transformation, and demonstrate that the enhanced alternative polyadenylation in proliferative conditions results not only in global 3' UTR shortening but also in enhanced premature cleavage in introns. Our results also indicate that E2F-mediated co-transcriptional regulation of 3'-end processing genes is one of the mechanisms that links enhanced alternative polyadenylation to proliferation.

## Background

Cleavage and polyadenylation are required for the maturation of most mRNA transcripts [1]. The pre-mRNA is cleaved approximately 10 to 30 nucleotides downstream of the polyadenylation signal (PAS) and an untemplated poly(A) tail is added to the transcript. The canonical PAS is AAUAAA, which appears in about 50% of the cleavage sites (CSs). More than 10 PAS variants have been documented, the most prevalent of which is AUUAAA [2,3]. In addition, various upstream and downstream auxiliary elements were found to regulate the efficiency of poly(A) site usage, prominent among them U-rich and GU-rich elements downstream of the cleavage sites [2]. Recently, using proteomics analysis, more than 90 proteins were shown to consist or physically interact with the pre-mRNA 3'-end processing machinery in human cells [4]. Important units in this machinery are the cleavage and polyadenylation specific factor (CPSF) complex, which interacts with the PAS, and the cleavage stimulation factor (CstF) complex, which interacts with the U/GU-rich downstream elements [5,6].

Recent studies have demonstrated that alternative cleavage and alternative polyadenylation (APA) are much more prevalent than was previously appreciated, and that they involve more than 50% of mammalian genes [7-9]. The number of genes reported to contain alternative cleavage and polyadenylation sites in their 3' UTR is steadily increasing, mainly due to the maturation of deep-sequencing techniques specifically adapted for mapping of transcript 3'-ends [7,8,10,11]. 3' UTRs carry high regulatory potential as they serve as a flexible platform for interactions between target transcripts and RNA-binding proteins and regulatory RNAs (mainly microRNAs (miRNAs)) [12,13]. Those interactions play major roles in regulating transcript stability, localization and translational efficiency [12,13]. APA modulates these modes of gene regulation by generating gene isoforms that differ in their 3' UTRs.

The functional significance of APA is largely unknown and is just beginning to emerge (recent reviewed in [14]). Recent reports linked APA to proliferation [15], development [8,16,17] and cancer [18]. Sandberg *et al. *[15] reported a general link between proliferation and 3' UTR shortening in multiple cell types and tissues. Mechanisms that underlie this broad 3' UTR shortening associated with increased proliferation are currently unknown. Mayr and Bartel [18] linked APA also to cancer and suggested that cellular transformation is also associated with broad 3' UTR shortening, over and above the association expected from their proliferative state. However, as these two studies were based on expression array data, whose capacity to study APA is limited, comprehensive analysis of APA during cellular proliferation and transformation transitions is still lacking.

Accumulating evidence indicates a dynamic interplay between polyadenylation and splicing. It was reported that approximately 20% of human genes have polyadenylation sites within their introns, most of which result in altered protein products [19]. A dynamic competition between the cleavage and splicing machineries was implied by the fact that intronic cleavage sites were more prevalent in larger introns with weaker 5' splice site signal. An additional insight into the interplay between these two processes was provided by a recent study that observed that knocking-down U1 small nuclear ribonucleoprotein (U1 snRNP) resulted in extensive induction of cleavage at numerous cryptypic polyadenylation sites within introns, suggesting that, under normal conditions, this factor protects against premature intronic cleavage [20].

In this study we set out to dissect and compare, on a transcriptomic-scale, APA events associated with proliferation and transformation. We applied a deep-sequencing method for mapping and quantification of polyadenylation sites to two human cellular systems, primary fibroblast BJ cells and non-transformed mammary epithelial MCF10A cells, each of them examined under proliferative, arrested and transformed states. In both cell lines, the proliferative state was strongly associated with enhanced APA, resulting not only in global 3' UTR shortening but also in enhanced premature cleavage at intronic polyadenylation sites. The link between 3' UTR shortening and proliferation was markedly stronger than its association with transformation. Furthermore, our results pinpoint E2F-mediated transcriptional co-regulation of genes that encode 3'-end processing proteins as one of the mechanisms underlying the association between enhanced APA and proliferation.

## Results

### Transcriptome-wide characterization of polyadenylation sites using 3'-Seq

**Figure 1:**
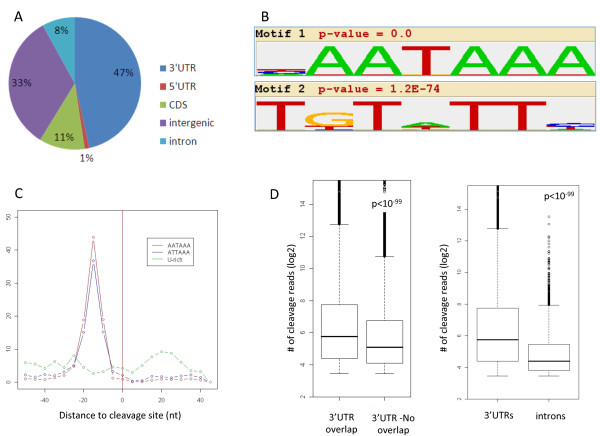
**Identification of poly(A) sites using 3'-Seq**. **(a) **Distribution of the cleavage sites (CSs) identified by 3'-Seq into genomic regions. **(b) **Enriched motifs identified in the region spanning ±50 nucleotides with respect to the CSs that mapped to the 3' UTR. The top scoring motif corresponds to the canonical PAS and the second top corresponds to the auxiliary U-rich motif. **(c) **Location distribution of the enriched signals with respect to the position of the mapped cleavage sites. **(d) **Comparison between the strength of 3' UTR CSs (estimated by the number of sequenced reads that support their call) that either overlapped or did not overlap polyA-DB records (left), and between CSs that mapped to 3' UTR or introns (right). CDS, coding sequence; nt, nucleotide.

We set out to characterize, on a transcriptomic scale, changes in APA that are associated with alternations in the proliferative state of cells. To this goal we adapted from Beck *et al. *[21] a deep-sequencing method, which we term 3'-Seq, that allows wide-scale identification of polyadenylation cleavage sites (poly(A) CSs), quantification of their usage, and their mapping at a nucleotide resolution (Materials and methods; Figure S1a-c in Additional file 1). We applied 3'-Seq to two human cellular systems, primary fibroblast BJ cells and non-transformed MCF10A epithelial cells, each examined under proliferative and arrested states as described below. A total of 43,468 poly(A) CSs were identified in the entire dataset (Additional file 2), showing as expected a significant enrichment for 3' UTRs (approximately 50% of the CSs were mapped to annotated 3' UTRs; Figure 1a). Motif analysis showed that the sets of CSs that mapped to 3' UTRs and introns were highly enriched for the major signals recognized by the cleavage machinery, namely the PAS and the auxiliary U-rich motifs (Figure 1b; Figure S1d in Additional file 1). Furthermore, the location distribution of these motifs peaked at the expected positions upstream and downstream of the putative CSs, respectively, demonstrating the precision of CS mapping obtained by 3'-Seq (Figure 1c; Figure S1d in Additional file 1). The sets of CSs that mapped to coding sequence and intergenic regions showed much reduced enrichment for the known cleavage signals, possibly indicating higher rates of false calls in these sets, and therefore they were excluded from subsequent analysis.

Next, we examined the overlap between the CSs identified in our experiment and the annotated sites recorded in polyA-DB [22]. While 40% of the 3' UTR CSs in our dataset overlapped polyA-DB records, only 3% of the intronic CSs did so. We observed that the set of 3' UTR CSs identified in our dataset that did not overlap polyA-db records were significantly weaker than those that did overlap, and that intronic CSs were, as a set, significantly weaker than 3' UTR sites (Figure 1d), showing the improved sensitivity of 3'-Seq in identification of weaker poly(A) sites.

### Association between APA and proliferation in primary fibroblast BJ cells

**Figure 2:**
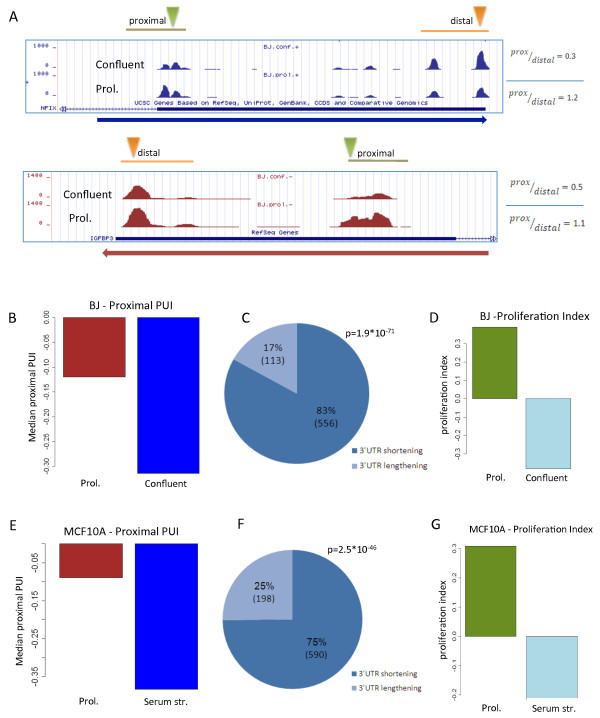
**Extensive 3' UTR shortening in proliferative cells**. **(a) **Two examples of transcripts that showed enhanced usage of proximal CSs in BJ proliferating cells compared to confluent ones. The blue and red plots correspond to genes encoded on the positive and reverse strands, respectively. Proximal and distal CSs are indicated by green and orange triangles, respectively. Ratios between proximal and distal peak levels are indicated to the right. **(b) **The proximal polyadenylation site usage index (proximal PUI) provides a global measure for the relative usage of proximal CSs in the examined conditions (Materials and methods). The transition from proliferation to confluent state resulted in a strong decrease in the proximal PUI. **(c) **A pie chart showing the distribution of transcripts that underwent significant change in CS usage in the transition from proliferation to confluent. In dark and light blue are the proportions of transcripts with shortened or lengthened 3' UTRs in the proliferative state, respectively. **(d) **The proliferative status of proliferating and confluent BJ cells was measured using a proliferation index, which is based on the relative expression of cell-cycle related genes. **(e-g) **Same as (b-d), but here proliferating and serum-deprived MCF10A cells were analyzed.

We first analyzed the 3'-Seq data recorded in human primary fibroblast BJ cells, either grown under normal proliferative conditions or induced to arrest by reaching a confluent state, and examined the effect of the proliferative status on APA. In these BJ samples, 3' UTR poly(A) CSs were mapped to 8,749 transcripts, of which 1,934 contained multiple CSs in their 3' UTRs. To detect global APA modulation associated with the change in BJ cells' proliferative state, we defined the 'proximal poly(A) site usage index' (proximal PUI), which serves as a global measure for the strength of usage of proximal 3' UTR poly(A) sites in each condition (Materials and methods). The transition of BJ cells from proliferation to confluence was accompanied by a marked decrease in the proximal PUI (that is, attenuated usage of proximal CSs), which reflected a global 3' UTR lengthening associated with this transition (Figure 2a,b). We next tested the transcripts for changes in CS usage (Materials and methods; Figure S1e,f in Additional file 1). Of the 1,934 transcripts with multiple 3' UTR CSs, 669 showed significant (*P *< 0.001) shift in CS usage in the comparison between proliferative and confluent cells. Remarkably, in 556 (83%) of these cases, the shift was towards increased usage of proximal CSs in the proliferating cells (*P *= 1.9 × 10^-71^, binomial distribution tail; Figure 2c), confirming, on a broader scale and with improved sensitivity, a previous report on the association between proliferation and 3' UTR shortening [15].

3'-Seq allows not only the identification of poly(A) sites, but also the quantification of gene expression levels (similar to standard RNA-Seq, the number of reads that align to a transcript provides an estimate for its expression level). Gene Ontology (GO) analysis showed that the set of genes that were repressed in the transition of BJ cells from the proliferative to confluent state were highly enriched for cell-cycle related functional categories, and the set of genes that were induced in this state were enriched for developmental and adhesion processes (Table S2a in Additional file 1). To obtain a quantitative measure of the proliferative status of the examined cells, we calculated for each condition a proliferation index, based on the relative expression levels of cell-cycle-related genes (Materials and methods). The proliferation indices calculated for the proliferating and confluent BJ cells reflected the strong decrease in proliferation in the transition to the confluent state (Figure 2d).

Next, we examined whether the change in CS usage was associated with any alterations in expression level. Under the assumptions that 3' UTRs mainly serve as a platform for miRNA-mediated regulation, and that miRNAs generally restrict target transcript levels, we hypothesized that 3' UTR shortening would result in increased expression of the affected transcripts due to removal of miRNA target sites. However, we did not observe that changes in poly(A) CS usage resulted in a significant effect on transcript level (Figure S2a in Additional file 1). We therefore conclude that the proliferative state of primary human BJ cells is associated with a widespread increase in the usage of proximal poly(A) CSs, resulting in 3' UTR shortening in hundreds of transcripts, but this induction of APA does not seem to have a significant global effect on the expression level of the targeted transcripts.

### Association between APA and proliferation in non-transformed epithelial MCF10A cells

Subsequently, we wished to generalize the results we obtained with BJ cells using a second cellular model, human non-transformed MCF10A epithelial cells, which were arrested by serum deprivation. In this dataset we identified 8,835 transcripts, of which 1,681 contained multiple CSs in their 3' UTRs. Here too, the proliferative state was associated with increased cleavage at proximal 3' UTR CSs, reflected by the higher value of the proximal PUI measure (Figure 2e). Furthermore, among the 1,681 transcripts with multiple CSs, 788 showed significant shift (*P *< 0.001) in CS usage in the comparison between serum-deprived and control MCF10A cells. Significantly, proliferation was strongly associated with a broad shortening of 3' UTRs: 590 transcripts (75%) showed enhanced usage of proximal CSs in the proliferating cells compared to the serum-starved ones (*P *= 2.5 × 10^-46^, binomial distribution tail) (Figure 2f). Gene expression analysis and calculation of the proliferation index demonstrated the strong decrease in proliferation that resulted from the deprivation of serum (Figure 2g; Table S2b in Additional file 1). Similar to BJ cells, changes in APA did not affect mRNA expression levels (Figure S2b in Additional file 1), reinforcing the conclusion that the strong APA modulation associated with proliferation does not have a major global effect on mRNA levels. Still, it is possible that only few genes show marked change in mRNA levels following APA, or that APA impacts global gene expression at regulatory layers downstream of mRNA production and stability (for example, mRNA localization and translation efficiency).

**Figure 3:**
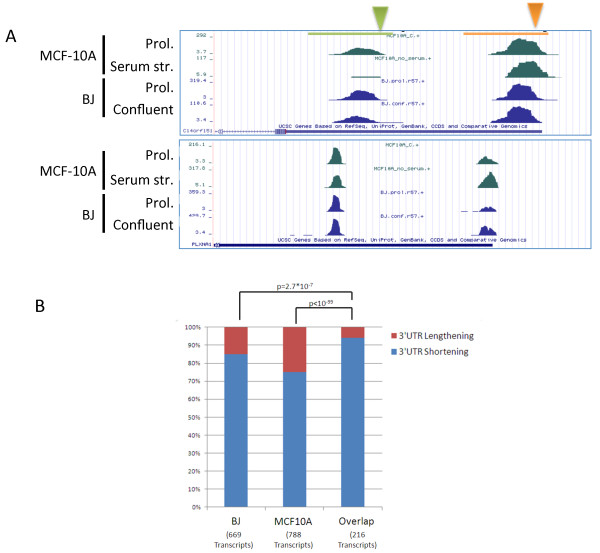
**Core set of genes with shortened 3' UTRs in proliferative state**. **(a) **Examples of transcripts with shortened 3' UTRs in the proliferative state compared to the arrested one in both BJ and MCF10A cells. Proximal and distal CSs are indicated by green and orange triangles, respectively. **(b) **The set of 216 transcripts that showed consistent change in 3' UTR length in both BJ and MCF10A cells was significantly enriched for genes with shortened 3' UTRs in the proliferative state (the increase from 83% and 75% to 94% is highly significant; *P*-values calculated using geometric tail).

Examination of the relationship between proliferation and APA using two different cellular models and two methods for modulating the proliferation state of the cells allowed us to identify a core set of transcripts that showed consistent change in 3' UTR CS usage in both systems. This core set contained 216 transcripts (in contrast to 68 transcripts that showed opposing tendencies in the change in 3' UTR length in the two systems; Additional file 3). The core set of 216 transcripts was significantly enriched for those that underwent 3' UTR shortening in proliferation: 203 transcripts (94%) in this set showed enhanced usage of proximal CSs in both proliferating BJ and MCF10A cells compared to their arrested counterparts, while only 13 (6%) showed 3' UTR lengthening in the proliferative state of both cellular systems (Figure 3a,b). The genes in this core set function in various biological processes and were not enriched for any particular functional category.

### Proliferation is associated with enhanced cleavage at intronic poly(A) sites

**Figure 4:**
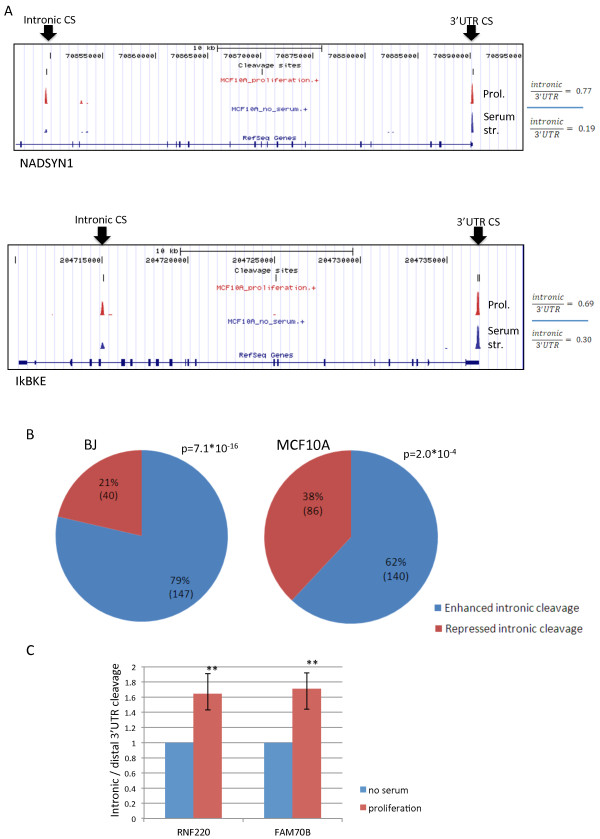
**Enhanced cleavage at intronic poly(A) sites in proliferation**. **(a) **Two examples of genes that showed significant enhancement of cleavage at intronic poly(A) sites in proliferative MCF10A cells compared to serum-starved ones. Ratios between intronic and 3' UTR peak levels are indicated to the right. **(b) **Global enhancement of cleavage at intronic sites was associated with proliferation in both BJ cells (left, proliferative compared to confluent cells) and MCF10A cells (right, proliferative compared to serum starved cells). **(c) **Enhanced cleavage at intronic sites in proliferation was validated using 3'-end qRT-PCR applied to intronic and 3' UTR distal poly(A) sites of RNF220 and FAM70B, measured in MCF10A under proliferative and serum-starved conditions. The figure shows means (± standard deviation) of three independent experiments (for both genes, ^**^P < 0.005; one-tailed *t*-test).

The enhanced usage of proximal 3' UTR cleavage sites in proliferative states suggests that the activity of the cleavage machinery is somehow augmented in proliferation. Therefore, we hypothesized that rates of premature cleavage at intronic poly(A) sites should also increase in proliferating cells. Some 8% (approximately 3,450 sites) of the cleavage sites in our 3'-Seq dataset mapped to introns (Figure 1a), and this set of sites showed marked features of authentic poly(A) sites (Figure S1d in Additional file 1). To test our hypothesis, we statistically examined the transcripts for which we mapped both intronic and 3' UTR CSs for significant shifts between the usage of these sites. Indeed, we found that in both the BJ and MCF10A cellular systems, the proliferative state was associated with significantly elevated rates of cleavage and polyadenylation at intronic sites (Figure 4a,b). We validated the proliferation-associated enhanced cleavage at intronic poly(A) sites using 3'-end quantitative RT-PCR (qRT-PCR; Materials and methods) applied to two target transcripts, RNF220 and FAM70B, probed in MCF10A cells under normal growth and serum starved conditions (Figure 4c).

### APA events during cellular transformation

**Figure 5:**
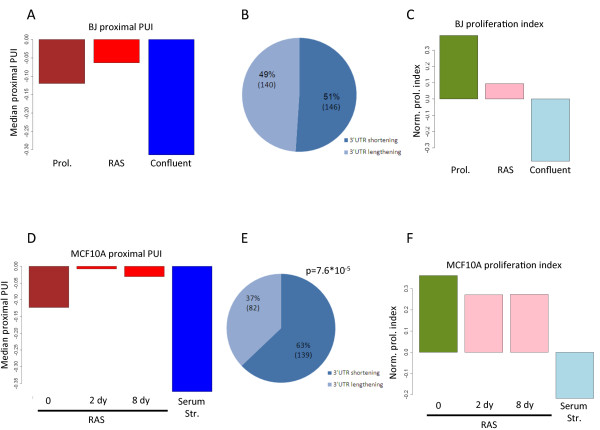
**Relationship between APA and transformation**. **(a) **Proximal PUIs calculated for proliferating, RAS-transformed and confluent BJ cells. **(b) **Distribution of APA events induced by RAS transformation according to net effect on transcript 3' UTR length. **(c) **Proliferation index calculated for proliferating, RAS-transformed and confluent BJ cells. **(d) **Proximal PUIs calculated for proliferating, RAS-transformed (2 and 8 days after RAS^G12V ^induction) and serum-starved MCF10A cells. **(e) **Distribution of APA events that were consistently induced by RAS transformation at both 2 days and 8 days, according to net effect on transcript 3' UTR length. **(f) **Proliferation index calculated for proliferating, RAS-transformed and serum-starved MCF10A cells.

Next, we used the BJ and MCF10A cellular systems to study the relationship between cellular neoplastic transformation and APA. BJ cells were induced to transform by ectopically expressing oncogenic-HRAS^G12V ^(in short RAS^G12V^) in cells that also expressed short hairpin RNA (shRNA) constructs targeting p53 and p16INK4A, two failsafe tumor suppressor genes in the oncogene-induced senescence pathway [23]. 3'-Seq was applied to transformed (3 days after RAS^G12V ^induction) and control proliferating cells. As with proliferation arrest, we observed many changes in APA in this system too. However, unlike the drastic tendency towards 3' UTR shortening that was associated with the proliferative state of BJ cells, the APA events that occurred during the transition of BJ cells into a transformed state were evenly distributed between shortening and lengthening of 3' UTRs (Figure 5a,b). Of note, the transition into the transformed state was accompanied by a mild decrease in proliferation as measured by the proliferation index (Figure 5c).

Analyzing APA associated with transformation of MCF10A cells led to a similar conclusion. We transformed MCF10A cells by ectopic induction of oncogenic RAS^G12V ^(which is sufficient, without any further genetic manipulation, for efficient transformation of MCF10A [24]), and harvested them at time points 0, 2 and 8 days following RAS^G12V ^induction. Figure S3 in Additional file 1 shows the efficient induction of RAS^G12V ^and its associated induction of transformation phenotype as measured by anchorage-independent growth of cells in soft agar. The intersection between the two time points after RAS induction identified 221 transcripts that showed significant shift in CS usage that was consistent in its direction (only 19 changes occurred in opposite directions). Out of these 221 transcripts, 139 (63%) showed a shift towards proximal CSs (Figure 5d,e). A slight decrease in the proliferation index was observed in RAS^G12V^-transformed MCF-10A cells (Figure 5f). Altogether, this cellular model also demonstrates that the 3' UTR shortening effect, which is associated with the transition from the arrested to proliferating state, is stronger than the 3' UTR shortening effect associated with the transition from the proliferative to transformed state.

### E2F transcriptional regulation of 3'-end processing genes

**Figure 6:**
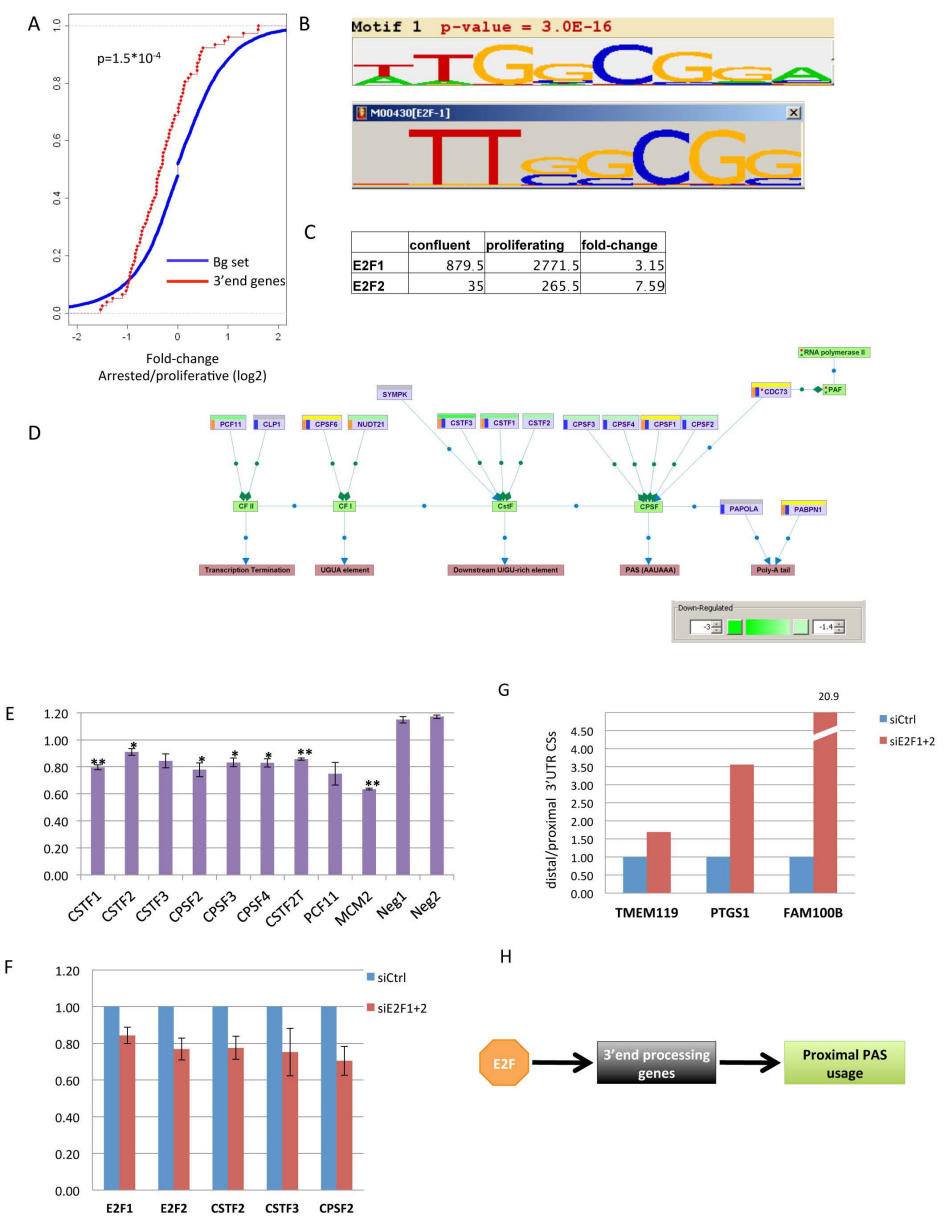
**E2F-mediated regulation of APA in proliferation**. **(a) **Comparison of the distribution of fold-change (in log2) in expression levels between confluent and proliferative cells calculated over two sets of genes: a target set that contained the 3'-end processing genes and a background set that contained all the rest of genes expressed in BJ cells (*P*-value calculated using Wilcoxon test). **(b) **The top scoring motif identified by *de novo *motif discovery analysis applied to the set of promoters of the 3'-end processing genes matched the binding signature of E2F (motif accession number in TRANSFAC DB: M00430). **(c) **Normalized expression levels (determined from the 3'-seq data) of E2F1 and E2F2 in proliferating and confluent BJ cells. **(d) **Schematic map of the core machineries of polyadenylation site recognition and 3'-end cleavage. In this map, nodes correspond to either protein-coding genes (violet nodes) or protein complexes (green nodes), and edges correspond to either regulatory links (blue edges) or association links between complexes and their members (green edges). The fold-change in expression in the transition from proliferating to confluent BJ cells is indicated by the horizontal bar at the top of each node (green corresponds to down-regulation, yellow to no-change and gray to gene not expressed in our dataset). Genes in this network whose promoter was found to be bound by either E2F1 [26] or E2F4 [27] are indicated by an orange and blue vertical bars to their left. (The map was created using the SPIKE knowledgebase of signaling pathways [36].) **(e) **Effect of knocking down E2F1 on promoter activity of eight 3'-end processing genes assessed using reporter assays. MCM2 served as positive control and an artificial p53 promoter was used as a negative one. *P < 0.05, **P < 0.01. Error bars represent SEM. **(f) **The effect of knocking down E2F1+2 on the expression levels of three core cleavage factors in BJ cells was examined using qPCR (results shown are mean ± standard deviation based on triplicates in the case of E2F1 and E2F2, and on five replicates for the three cleavage transcripts). In all cases, the reduction in transcript level is statistically significant (*P *< 0.01; one-tail *t*-test)). **(g) **The effect of knocking down E2F1+2 on the relative usage of 3' UTR proximal and distal CSs in three transcripts that showed enhanced usage of the proximal CSs in proliferation was examined using 3'-qPCR (Materials and methods). In all three cases examined, reducing E2F levels increased the relative cleavage at the distal site (namely, reduced the cleavage at the proximal one). Results shown are based on duplicates; in all cases, *P *< 0.05, one-tail *t*-test). **(h) **A schematic model illustrating the E2F-mediated regulation of mRNA 3'end processing enzymes and its effect on APA.

The mechanisms underlying the association between enhanced APA and proliferation are currently unknown. Seeking such mechanisms, we first examined the change in expression level of a set of 94 genes that encode proteins that function in transcript 3'-end processing [4]. Intriguingly, the transition of BJ cells from proliferation to confluence resulted in a significant decrease in the expression of this set of genes (*P *= 0.00015; Figure 6a). To further corroborate the observation that the transition of BJ cells from the proliferative to arrested state is associated with decreased levels of 3'-end processing genes, we carried out an independent RNA-seq experiment applied to proliferating and serum-starved arrested BJ cells. Here, too, the expression level of this set of genes was significantly down-regulated in the arrested state (Figure S4a in Additional file 1). In addition, down-regulation of this set of genes, albeit at a lower statistical significance (*P *= 0.024), was observed also in the transition of MCF10A cells from the proliferative to arrested state (Figure S4b in Additional file 1).

The co-repression observed for the set of transcripts encoding the proteins of the 3'-end processing machinery suggested their transcriptional co-regulation. Hence, we next searched their promoter regions for over-represented *cis*-regulatory motifs. Interestingly, the top-scoring motif detected by *de novo *motif analysis matched the binding signature of the E2F family (Figure 6b), which is one of the master regulators of the transcriptional network associated with cell cycle progression [25]. As expected, the expression levels of E2F1 and E2F2 significantly decreased in the transition from proliferative to confluent state (by 3- and 7.5-fold, respectively; Figure 6c). To further support the regulation of the 3'-end processing machinery by E2F, we searched for publicly available datasets that profiled E2F-chromatin interactions on a genomic scale. ChIP-Seq analyses were recently applied to the E2F1 [26] and E2F4 [27] members of this family. While E2F4 mainly acts as transcriptional repressor, to a large extent it shares its binding signature and target genes with E2F1-3, which are transcriptional activators [25]. We found that many promoters of key 3'-end processing genes were reported to be bound by E2F1 and/or E2F4 (Figure 6d). To demonstrate more directly a role for E2F in the regulation of 3'-end processing genes, we cloned promoter regions of eight core components of the cleavage machinery upstream of a luciferase gene in the pGL3 reporter construct, and examined the effect of knocking-down E2F1 on promoter activity. The promoter region of MCM2, a potent E2F target gene [28], was used as a positive control, and a reporter containing an artificial p53 promoter was used as negative ones. In six out of the eight 3'-end processing gene promoters examined, silencing E2F1 resulted in a statistically significant reduction in promoter activity (Figure 6e), and in the other two cases reduction in promoter activity was also observed, but did not reach the statistical cutoff of 0.05. (The effect of E2F1-kd was mild on all the targets examined, including the positive control, MCM2, probably due to redundancy between E2F members and residual E2F1 activity remaining after knocking it down.) Next, we examined the effect of knocking down E2F1 and E2F2 on the expression of three core cleavage factors: CSTF2, CSTF3 and CPSF2. In all these cases we confirmed that knocking down E2F resulted in deceased levels of the cleavage factors (Figure 6f). Last, we selected three target genes that showed highly significant induction of cleavage at 3' UTR proximal CSs in proliferation, and examined the effect of knocking down E2F1 and E2F2 on the relative usage of the proximal and distal sites of these transcripts. Reducing the levels of E2F indeed resulted in weakened cleavage at the proximal sites of these transcripts (Figure 6g).

Taken together, our results indicate that E2F-mediated co-regulation of genes that function in recognition and cleavage of poly(A) sites contributes to the link between increased cellular proliferation and enhanced usage of proximal CSs (Figure 6h).

## Discussion

Previous studies that reported the association between APA and proliferation, transformation and development were mainly based on microarray measurements [15-18]. Consequently, these analyses were limited to those 3' UTRs that were covered by array probes both downstream and upstream of proximal CSs (a setting that allows per gene comparison between the expression level of the short and long 3' UTR isoforms), to pre-defined poly(A) sites inferred from EST databases, and to 3' UTRs with exactly two poly(A) sites. Here, we applied 3'-Seq, a deep-sequencing technique specifically designed for identification and quantification of poly(A) sites. Applying this technique to human cellular systems in proliferative and arrested states enabled us to examine APA events associated with proliferation at a much improved scale and sensitivity. Our analysis identified hundreds of previously uncharacterized poly(A) sites and dozens of novel APA events that occur during the transitions between the proliferative, arrested and transformed states. Moreover, beyond confirming on a broader scale and with improved resolution the global 3' UTR shortening associated with cellular proliferation, the two very different cellular models used in our study allowed the identification of a core set of 216 transcripts subjected to this mode of regulation. The statistical test that identifies changes in CS usage is inherently less sensitive to lowly expressed transcripts (covered by lower number of sequenced reads; Figure S5 in Additional file 1), and therefore it is very likely that many more transcripts undergo 3' UTR shortening in proliferation. In agreement, random partition of our dataset into subsets of increasing size showed that the number of identified APA events in our dataset was not reaching saturation (data not shown). Hence, the identification of APA events on lowly expressed genes requires increase in sequencing depth.

The mechanism underlying the enhanced APA associated with proliferation is unknown. Previous reports observed that embryonic development is associated with progressive lengthening of 3' UTRs, which is correlated with decreased levels of the set of genes encoding the 3'-end processing machinery [17,29]. This observation indicated that 3' UTR lengthening is likely caused by weakening of mRNA polyadenylation activity. Similarly, our analysis identified elevated expression of the set of genes encoding the 3'-end processing machinery in proliferating BJ cells compared to arrested ones. The collective induction of this set of genes could explain the extensive 3' UTR shortening observed in proliferation. Mechanistically, the co-induction of these genes in proliferation suggested their co-transcriptional regulation. In accordance, we identified significant over-representation of the E2F binding motif in the promoters of this set of genes. Additionally, we noted a physical interaction of members of the E2F family with many promoters in this set of genes. We demonstrated empirically a role for E2F1 in regulating key genes in this set, and showed that reducing E2F levels decreased cleavage at proximal 3' UTR CSs of selected target transcripts. Thus, our results suggest that elevated levels of E2F members in proliferative states lead to transcriptional activation of key 3'-end processing genes, resulting in enhanced 3' cleavage and polyadenylation activity, which leads to widespread 3' UTR shortening.

Furthermore, our results show that the enhanced APA associated with proliferation results not only in global 3' UTR shortening but also in increased rates of premature cleavage at intronic poly(A) sites. This observation also indicates augmentation of mRNA polyadenylation activity in proliferation. A previous report suggested a dynamic competition between the splicing and polyadenylation apparatuses [19], and recently a mechanism that suppresses premature cleavage and polyadenylation from cryptic polyadenylation signals located in introns was documented [20]. Our results suggest that E2F-mediated elevation in expression of 3'-end processing genes in proliferation causes augmented cleavage at both intronic and 3' UTR proximal CSs. Yet, it is clear that many more factors are involved in APA regulation. Using a RNA interference screen, we recently identified PABPN1 as an APA-regulator [30]. We found that knocking-down PABPN1 resulted in global enhancement of cleavage at proximal 3' UTR CSs, reminiscent of the effect observed in the transition from the arrested to proliferative state. We are currently studying the possible role for PABPN1 in regulating APA in proliferation.

3' UTR shortening was previously associated with both proliferation [15] and oncogenic transformation [18], linking APA to human cancer. However, the relative contribution of the enhanced proliferative capacity and the transformed phenotype to the global shortening of 3' UTRs had not previously been empirically assessed. We employed a very controlled setup using two very different cellular systems manipulated to change their status from the proliferative to arrested or transformed states. Both our systems, BJ and MCF10A cells, led to the same observation: increased usage of proximal poly(A) sites was more strongly associated with proliferation than with transformation. Recently, Fu *et al. *[11] applied a deep-sequencing method to compare APA between two breast cancer cell lines, MCF7 and MB231, and the non-transformed MCF10A cells. They found opposing tendencies in the change in 3' UTR length: while MCF7 cells showed mainly 3' UTR shortening compared to MCF10A cells, MB231 cells showed extensive 3' UTR lengthening, further indicating the complexity of APA regulation and demonstrating its dependence on cellular context beyond the effect of proliferative and transformation states.

3' UTRs carry high regulatory potential by serving as major docking platforms for RNA-binding proteins and miRNAs. As miRNAs are believed to play mainly a repressive role, it could have been expected that the global loss of miRNA target sites stemming from the extensive shortening of 3' UTRs in proliferation would result in increased expression of the affected transcripts. However, in the two cellular systems that we examined we did not find any global effect for the 3' UTR shortening/lengthening on expression level of the target genes. Similar to our observation, the global 3' UTR shortening detected in response to activation of immune cells [15] did not result in a significant effect on transcript expression level. It is possible that the enhanced APA elicits strong changes in mRNA levels of only a few genes and/or a more significant effect on translation and mRNA subcellular localization of the target genes, as 3' UTRs play major roles in these two layers of gene regulation as well.

It is also relevant to note that our conclusion on global 3' UTR shortening in proliferation is based on the observation that, in these conditions, the levels of proximal poly(A) sites were globally elevated relative to the level of distal ones. Yet, such a global increase in the relative levels of proximal poly(A) sites could, in principle, stem not only from enhanced APA, but also from some widespread mRNA destabilization that preferentially affects the longer isoforms (for example, global induction of miRNA activity in proliferation compared to arrested cells). A model that argues for a global destabilization of longer isoforms as a major contributor to the relative increase in the level of proximal CSs would predict that the transcripts that undergo 3' UTR shortening should show overall decreased expression. However, no evidence for such an effect was observed in our data. Furthermore, the elevated level of the set of genes encoding 3'-end processing factors and the enhanced cleavage at intronic poly(A) sites observed in the proliferative states strongly argue that enhanced polyadenylation and cleavage activity, and not stability modulation of the target transcripts, indeed comprise the underlying mechanism for the relative increase in the levels of the proximal CSs.

## Conclusions

Our knowledge on the modulation of APA in various biological conditions is just beginning to grow, and this layer of gene regulation is anticipated to be involved in many processes relevant to human disease. Yet, the functional significance of APA is still unclear. What is the objective of the program that leads to the extensive 3' UTR shortening in cellular proliferation? And on the other hand, what is the biological significance of the broad 3' UTR lengthening associated with differentiation? These are still open questions that call for further investigations.

## Materials and methods

### Cell culture

Primary BJ foreskin fibroblasts containing expression constructs for the ecotropic receptor and the human telomerase gene (hTERT), BJ-EHT, were either maintained in a cycling state or contact inhibited by growing them to confluence in DMEM (Gibco, Bleiswijk, The Netherlands) supplemented with 10% fetal calf serum and antibiotics. BJ-EHT/p53kd/p16kd/RAS^GV12^ER cells expressing shRNA constructs targeting p53 and p16INK4A and 4-hydroxy-tamoxifen (4-OHT)-inducible oncogenic HRAS^G12V ^were cultured for 3 days in the presence of 100 nM 4-OHT to transform the cells. These cell lines are described in detail in [23]. BJ-EHT cells were transfected in a final concentration of 50 nM small interfering RNAs targeting E2F1 (targeting sequence 5′-GGCCCGAUCGAUGUUUUCC-3′) and E2F2 (targeting sequence 5′-GACUCGGUAUGACACUUCG-3′) using Dharmafect reagent (Dharmacon, Lafayette, Colorado, USA).

Non-transformed mammary epithelial MCF10A cells were either cultured in DMEM:F12 Ham's medium (Sigma, Zwijndrecht, The Netherlands) 1:1, supplemented with 10% fetal calf serum (Gibco), insulin (10 μg ml^-1^; Sigma), hydrocortisone (0.5 μg ml^-1^) and epidermal growth factor (20 ng ml^-1^; Peprotech, New Jersey, USA) or serum starved with DMEM:F12 containing no supplements for 48 hours. To transform the MCF10A cells, we transduced them with a retroviral vector expressing RAS^G12V^ER, and cultured them for 2 and 8 days in the presence of 100 nM 4-OHT.

U2OS cells were cultured in DMEM (Gibco) supplemented with 10% fetal calf serum and antibiotics.

### Construction of 3'-Seq libraries

Schematic representation of the 3'-Seq method is provided in Figure S1a in Additional file 1. Briefly, total RNA was extracted from either BJ or MCF-10A cells described above using the RNeasy Mini kit from Qiagen (Venlo, The Netherlands). mRNA was isolated with the Oligotex mRNA kit from Qiagen and 500 to 600 ng of the isolated mRNA was heat fragmented for 5 minutes at 70°C. The fragmented mRNA was then converted to single-stranded cDNA using SuperScript III reverse transcriptase (Invitrogen, Bleiswijk, The Netherlands) and a P7-t25-vn oligo-dT primer (5'-CAAGCAGAAGACGGCATACGAGATTTTTTTTTTTTTTTTTTTTTTTTTVN-3') according to the manufacturer's instructions. This was followed by second-strand cDNA synthesis (Invitrogen) and end-repair using T4 DNA polymerase, T4 polynucleotide kinase (NEBNext End Repair Enzyme Module, New England Biolabs, New England, USA) and Klenow DNA polymerase (Invitrogen). After purification with the QIAquick PCR Purification Kit (Qiagen), 1 µl of 45 µM annealed P5-splinkerette (forward, 5'-ATGATACGGCGACCACCGAGATCTACACTCTTTCCCTACACGACGCTCTTCCGATCT-3'; reverse, 5'-AGATCGGAAGAGCGTCGTGTAGGGTTTTTTTTTTCAAAAAAA-3') was ligated overnight at 16°C to the cDNA. The splikerette-ligated cDNA was then size-selected for approximately 220 bp long cDNA fragments using the E-Gel iBase Power System from Invitrogen. The final 3'-Seq libraries were then generated by PCR amplification of the linker-ligated cDNA with P5 and P7 primers. After a last purification step the 3'-Seq libraries were sequenced on the Illumina Genome Analyzer (BJ proliferation, confluent and RAS-transformed samples) or the Illumina HiSeq system (all MCF10A samples and technical repeats of the BJ proliferating and confluent samples).

### Analysis of 3'-Seq data

Sequenced reads were aligned against the human genome (hg18) using Bowtie [31]. Up to two mismatches were allowed in the reads' seed region (the reads' first 28 nucleotides). To allow the alignment of reads that span poly(A) cleavage sites and therefore contain the start of the untemplated poly-A tail, Bowtie's -e parameter was increased to tolerate mismatches in all bases after the seed region. Only uniquely mapped reads were used in subsequent analysis. Numbers of total and uniquely mapped reads are provided in Table S1 in Additional file 1. Wig files that summarize read coverage along the chromosomes (normalized to 10 million mapped reads) and raw sequence files are available at the Gene Eexpression Omnibus (accession number GSE33592). To map reads to genes and genomic regions (for example, 3' UTRs, introns, coding sequences, and so on), gene coordinates and annotations were extracted from the human Ensembl-Gene table of the UCSC browser [32]. To cover novel cleavage sites located downstream of current transcript 3'-end annotations, we extended the 3' UTR of each transcript by 1,000 bp.

### Mapping and characterization of poly(A) cleavage sites

In order to identify poly(A) CSs we searched for uniquely mapped reads that contained untemplated stretches of As (Figure S1b in Additional file 1). To reduce false calls that stem from priming of the oligo-dT primer to internal A-rich regions within transcripts, we required that, to support a CS call, reads should contain a stretch of at least eight As and at least five of the first eight As in the stretch should mis-match the corresponding bases on the transcript reference sequence (Figure S1c in Additional file 1). The location of the cleavage was taken as the location where the untemplated A stretch started. Since the location of the CSs often fluctuated around a major site, for each gene and sample we calculated a 'poly(A) CS profile', which recorded the number of reads supporting a cleavage at each position along the transcript (Figure S1e in Additional file 1). We considered the local maxima of these CS profiles as the CS locations, and required spacing of at least 50 nucleotides between consecutive CSs (in case of lower spacing between CSs, the stronger, that is, the one supported by a higher number of reads, was chosen). Only CSs supported by at least ten reads were considered in subsequent analyses.

### Differential usage of poly(A) cleavage sites

In order to identify significant shifts in 3' UTR poly(A) CS usage between the examined conditions, we first identified regions of 'read peaks' along transcripts' 3' UTRs. For this goal we divided each chromosome into 5-nucleotide bins, and counted how many reads started at each bin. To define peak boundaries that are common to all analyzed samples in a dataset, we took the union of the bin counts and calculated the maximum count at each bin over the samples. Peaks were defined as intervals of consecutive bin runs with maximum read count of least three reads (Figure S1e in Additional file 1). Peaks with a distance below 60 nucleotides were merged. Next, we tested each transcript that contained more than one 3' UTR CS for relationships between peak level and cellular state by chi-square test and using 0.001 as the significance cutoff (Figure S1f in Additional file 1). All tests were carried out using R. For transcripts that showed significant change in CS usage, in order to determine if the net shift was towards proximal or distal sites, we calculated, per transcript and condition, a weighted mean of CS index (weighting each CS (j) by its reads coverage (w_j_)):

$$\left(CS\text{\_}J\right)=\overset{\pi }{\underset{j=1}{\sum }}w_{j}*j$$

A decrease in <*CS_J*> indicates 3' UTR shortening in the corresponding condition and vice versa.

### Poly(A) site usage index

We defined the PUI in order to measure the relative usage of each 3' UTR poly(A) CS within a transcript. For a given transcript with *N *3' UTR cleavage sites, the PUI of the j-th cleavage site is defined by:

$$PUI_{j}=log_{2}\left(\frac{E_{j}}{\left(E\right)}\right);j=1..N$$

where *E_j _*is the level of the peak associated with the j-th cleavage site, and <*E*> is the geometric mean of the levels of all the peaks associated with the *N *cleavage sites. (By definition, the proximal PUI of a transcript in a certain condition is *PUI_j = 1_*.) In each condition, we calculated the distribution of proximal PUIs, and took its median as a global measure for the usage of proximal CSs in the corresponding samples. Global 3' UTR shortening in a certain condition is reflected by increase in its median proximal PUI.

### Gene expression analysis

As an estimate for expression level in each sample we took for each gene the total number of reads mapping to its 3' UTR. Quantile normalization and lower bound of expression level of five reads (to avoid division by 0) were applied before comparing expression levels between samples. GO enrichment analysis was carried out using GOrilla [33]. All expression analyses, except the one presented in Figure S4a in Additional file 1, were based on the 3'-Seq data. The analysis shown in Figure S4a in Additional file 1 is based on standard RNA-Seq applied to BJ cells either grown under normal proliferative conditions or deprived of serum for 48 hours. RNA-Seq was carried out using standard protocols and used Illumina's HiSeq platform. Reads were mapped to the canonical set of human transcripts, and expression levels were estimated using RPKM (reads per kilobase of exon model per million mapped reads) measures.

### *De novo *motif discovery

Sequences flanking ±50 nucleotides relative to the poly(A) CSs were extracted from the genome using Galaxy [34]. *De novo *motif discovery analysis was done using AMADEUS [35], and was applied to either CS regions or to the set of promoters of 94 genes encoding proteins consisting or physically interacting with the 3'-processing machinery [4]. Promoter regions were taken as -1,000 to +200 bp relative to the annotated transcription start sites. The set of promoters of all annotated human genes served as a background set for the analysis.

### Proliferation index

As a measure that reflects the proliferation status of cells under different conditions, we defined the proliferation index in each condition as the (log2) ratio between the median expression level of cell-cycle-related genes (genes assigned to the GO 'cell-cycle' functional category) and the overall median expression level in that condition.

### Promoter activity assays

U2OS cells were co-transfected with 450 ng of pRS or pRS-E2F1^KD ^in combination with 50 ng luciferase reporter and 5 ng renilla plasmid using polyethylenimine. Three days after transfection, luciferase assays were performed in accordance with the manufacturer's instructions (Dual Luciferase system; Promega, Utrecht, The Netherlands).

### qRT-PCR and 3'-end qRT-PCR

Total RNA was extracted using Trizol reagent (Invitrogen). cDNA was synthesized using Superscript III reverse transcriptase (Invitrogen) according to the manufacturer's instructions. Quantitative RT-PCR was performed with Light Cycler 480 Sybr Green I Master mix (Roche Applied Science, Almere, The Netherlands) and the Chromo 4 Real Time PCR Detector (Bio-Rad Laboratories, Providence, Rhode Island, USA). Primer sequences were: CSTF2, forward 5'-ATCCTTGCCTGCGAATGTCC-3', reverse 5'-GGGTGGTCCTCGGCTCTC-3'; CSTF3, forward 5'-TGAAGTGGATAGAAAACCAGAATACCC-3', reverse 5'-TGGAAACAGATAGGAGGAGGGAGA-3'; CPSF2, forward 5'-CTTTGGAACCCTTGCCACCT-3', reverse 5'-TGCGGACTGCTACTTGATTGTTG-3'; E2F2, forward 5'-ACTGGAAGTGCCCGACAGGA-3', reverse 5'-GGTGGAGGTAGAGGGGAGAGG-3'.

For 3'-end qRT-PCR, 2.5 μg of total RNA was heat fragmented with fragmentation buffer (Ambion, Bleiswijk, The Netherlands) for 5 minutes at 70°C. The fragmented total RNA was precipitated and the first strand of cDNA was synthesized as described above using a P7-t25-vn oligo-dT primer (5'-AAGCAGAAGACGGCATACGAGATTTTTTTTTTTTTTTTTTTTTTTTTVN-3'). qRT-PCR was performed as described above using P7-primer and the following gene-specific forward primers: β-actin distal forward 5'-CAGCCAGGGCTTACCTGT-3'; RNF220 intron forward 5'-TTTGGGTGGGGAAATGGAAT-3'; RNF220 distal forward 5'-CCTGTGGTGTGATGCTGTGTCT-3'; FAM70B intron forward 5'-CTCTGGCTCTGGGCTTCCC-3'; FAM70B distal forward 5'-GTTGATGCCCCCTGTGTTTG-3'; FAM100B proximal forward 5'-CAGGAGTTTTTCAGGCAAGTTTTTC-3'; FAM100B distal forward 5'-AGTGGAGAGCCTGCCTTTGG-3'; PTGS1 proximal forward 5'-GGCAAGGAAGTGGGGTGTTC-3'; PTGS1 distal forward 5'-CCTGCTAGTCTGCCCTATGGATTT-3'; TMEM119 proximal forward 5'-CCCTGGCAACATTGTGAGACC-3'; TMEM119 distal forward 5'-TCTCCCCCATCCCTCCATCT-3'.

## Abbreviations

4-OHT, 4-hydroxy-tamoxifen; APA, alternative polyadenylation; bp, base pair; CPSF, cleavage and polyadenylation specific factor; CS, cleavage site; DMEM, Dulbecco's modified Eagle's medium; GO, Gene Ontology; miRNA, microRNA; PAS, polyadenylation signal; PCR, polymerase chain reaction; poly(A) CS, polyadenylation cleavage site; PUI, poly(A)-site usage index; qRT-PCR, quantitative RT-PCR; shRNA, short hairpin RNA; UTR, untranslated region.

## Authors' contributions

RE performed all the bioinformatics analyses. JD performed most of the experimental work. GvH designed the 3'-Seq protocol. MJ carried out the 3'-Seq experiments. MS and JAFOV provided technical assistance. RA conceived and supervised the project. RE and RA wrote the manuscript.

## Competing interests

The authors declare that they have no competing interests.

## Supplementary Material

Additional file 1 **PDF file containing all supplementary figures, their legends and supplementary tables**. Click here for file

Additional file 2 **Excel file containing the coordinates of the cleavage sites identified by our 3'-Seq experiments**. Click here for file

Additional file 3 **Excel file containing information on the core set of 216 transcripts that were subjected to APA modulation in the transition from proliferation to the arrested state in both BJ and MCF10A cells**. Click here for file
