# Supplementary material for: E2F mediates enhanced alternative polyadenylation in proliferation
Source: Genome Biol. 2012 Jul 2;13(7):R59. doi: 10.1186/gb-2012-13-7-r59 (PMC3491381; doi:10.1186/gb-2012-13-7-r59)
Supplement: Additional file 1 — PDF file containing all supplementary figures, their legends and supplementary tables. [file gb-2012-13-7-r59-S1.PDF]

A

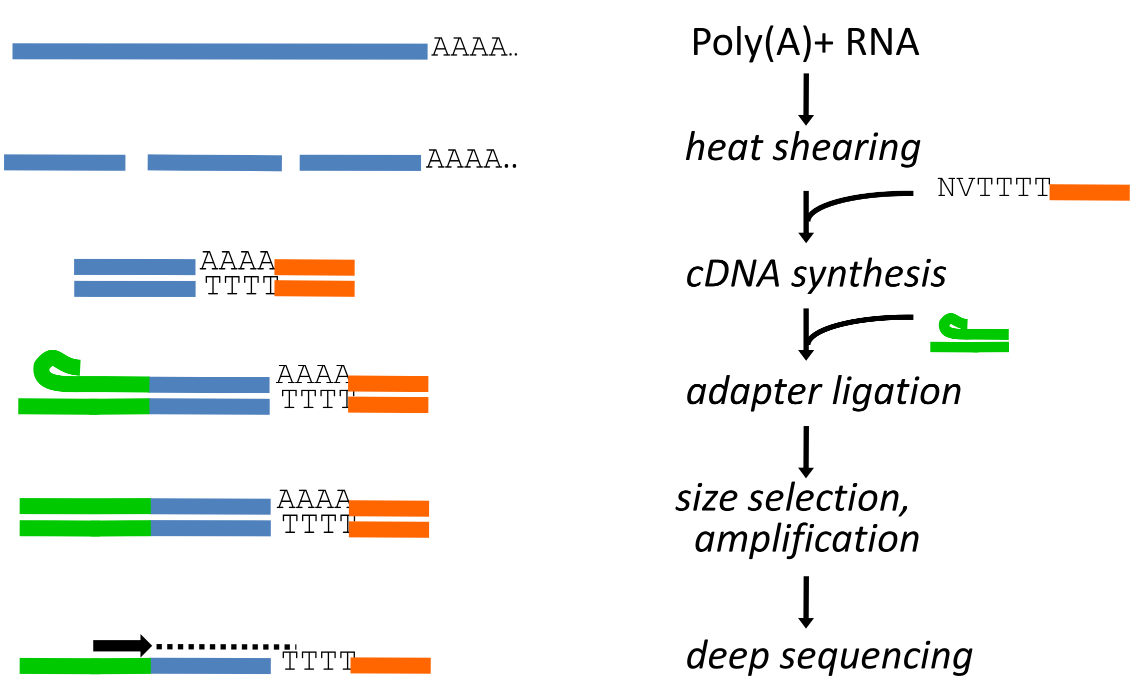

AAAA..
Poly(A)+ RNA
AAAA..
heat shearing
NVTTTT
AAAA
TTTT
cDNA synthesis
AAAA
TTTT
adapter ligation
AAAA
TTTT
size selection, amplification
TTTT
deep sequencing

Figure S1

B

```
  ATGAGGAGAGTGCTAGTTCATGTGTTCTCCATTCTTGTGAGCATCCTAATAAATCTGTTCCATTTTGATGACAGAAAAAA
  ATGAGGAGAGTGCTAGTTCATGTGTTCTCCATTCTTGTGAGCATCCTAATAAATCTGTTCCATTTTGATGACAGAAAAAA
    GAGGAGAGTGCTAGTTCATGTGTTCTCCATTCTTGTGAGCATCCTAATAAATCTGTTCCATTTTGATGACAGAAAAAAAA
      GGAGAGTGCTAGTTCATGTGTTCTCCATTCTTGTGAGCATCCTAATAAATCTGTTCCATTTTGATGACAGAAAAAAAAAA
              CTAGTTCATGTGTTCTCCATTCTTGTGAGCATCCTAATAAATCTGTTCCATTTTGACGAAAAAAAAAAAAAAA
                       GTGTTCTCCATTCTTGTGAGCATCCTAATAAATCTGTTCCATTTTGATGACTGAAAAAAAAAAAA
                         GTTCTCCATTCTTGTGAGCATCCTAATAAATCTGTTCCATTTTGATGACAGAAAAAAAAAAAAA
                         GTTCTCCGTTCTTGTGAGCATCCTAATAAATCTGTTCCATTTTGATGACAGAAAAAAAAAAAAAA
                            CTCCATTCTTGTGAGCATCCTAATAAATCTGTTCCATTTTGATGACAAAAAAAAAAAAAAAAA
                            CTCCATTCTTGTGAGCATCCTAATAAATCTGTTCCATTTTGATGACTAAAAAAAAAAAAAAAA
                               CATTCTTGTGAGCATCCTAATAAATCTGTTCCATTTTGATGACAGAAAAAAAAAAAAAAAAAA
                               CATTCTTGTGAGCATCCTAATAAATCTGTTCCATTTTGATGACAGAAAAAAAAAAAAAAAAAA
ACATGAGGAGAGTGCTAGTTCATGTGTTCTCCATTCTTGTGAGCATCCTAATAAATCTGTTCCATTTTGATGACAGAATGGCAGTGCCTGATTC
```

PAS

Poly(A) site

C

```
     GGGACAAAGTTCGAGAAGGGCCCCGTTCCCGATCAAGGTCCCGTGACCGCCGCCGCAAGGAACGTGCGAGGTCTAAAAAA
      GGACAAAGTTCGAGAAGGGCCCCGTTCCCGATCAAGGTCCCGTGACCGCCGCCGCAAGGAACGTGCGAAGTCTAAAAAAA
       GACAAAGTTCGAGAAGGGCCCCGTTCCCGATCAAGGTCCCGTGACCGCCGCCGCAAGGAACGTGCGAAGTCTAAAAAAAA
        ACAAAGTTCGAGAAGGGCCCCGTTCCCGATCAAGGTCCCGTGACCGCCGCCGCAAGGCACGTGCGAAGTCCAAAAAAAAA
         CAAAGTTCGAGAAGGGCCCCGTTCCCGATCAAGGTCCCGTGACCGCCGCCGCAAGGAACGTGCGAAGTCCAAAAAAAAAA
           AAGTTCGAGAAGGGCCCCGTTCCCGATCAAGGTCCCGTGACCGCCGCCGCAAGGAACGTGCGAAGTCTAAAAAAAAAAAA
            AGTTCGAGAAGGGCCCCGTTCCCGATCAAGGTCCCGTGACCGCCGCCGCAAGGAACGTGCGAAGTCTAAAAAAAAAAAAA
             GTTCGAGAAGGGCCCCGTTCCCGATCAAGGTCCCGTGACCGCCGCCGCAAGGAACGTGCGAAGTCTAAAAAAAAAAAAAA
              TTCGAGAAGGGCCCCGTTCCCGATCAAGGTCCCGTGACCGCCGCCGCAAGGAACGTGCGAAGTCTAAAAAAAAAAAAAAA
                CGAGAAGGGCCCCGTTCCCGATCAAGGTCCCGTGACCGCCGCCGCAAGGAACGTGCGAAGTCTAAAAAAAAAAAAAAAAA
                 GAGAAGGGCCCCGTTCCCGATCAAGGTCCCGTGACCGCCGCCGCAAGGAACGTGCGAAGTCTAAAAAAAAAAAAAAAAAA
                  AGAAGGGCCCCGTTCCCGATCAAGGTCCCGTGACCGCCGCCGCAAGGAACGTGCGAAGTCGAAAAAAAAAAAAAAAAAAA
                     AGGGCCCCGTTCCCGATCAAGGTCCCGTGACCGCCGCCGCAAGGAACGTGCGAAGTCTAAAAAAAAAAAAAAAAAAAAAA
GGATCGGGACAAAGTTCGAGAAGGGCCCCGTTCCCGATCAAGGTCCCGTGACCGCCGCCGCAAGGAACGTGCGAAGTCTAAAGAAAAGAAGAGTGAGAAGAAAGA
```

Figure S1

D

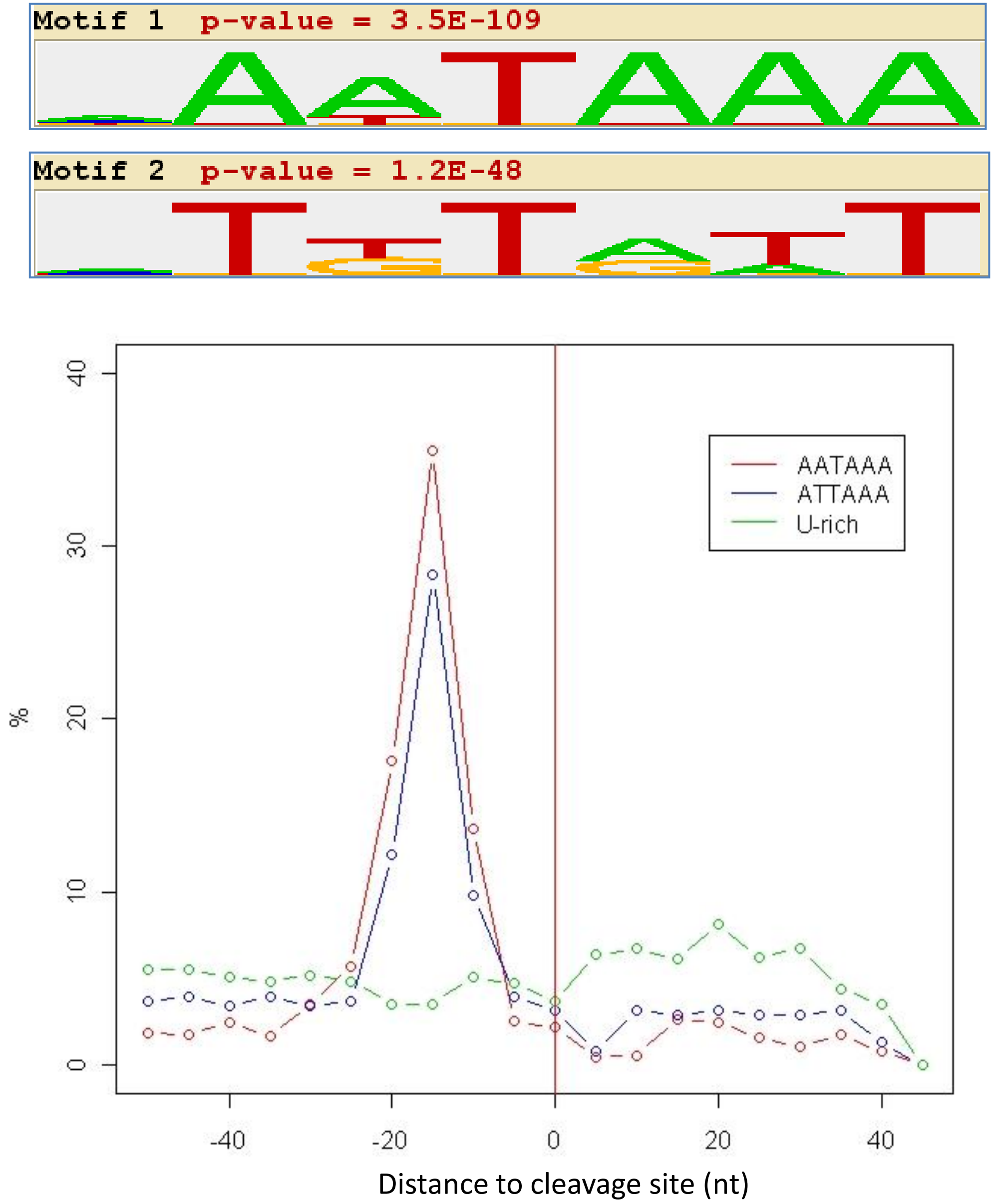
Motif 1 p-value = 3.5E-109
Motif 2 p-value = 1.2E-48
AATAAA
ATTAAA
U-rich
%
0
10
20
30
40
-40
-20
0
20
40
Distance to cleavage site (nt)

Figure S1

E

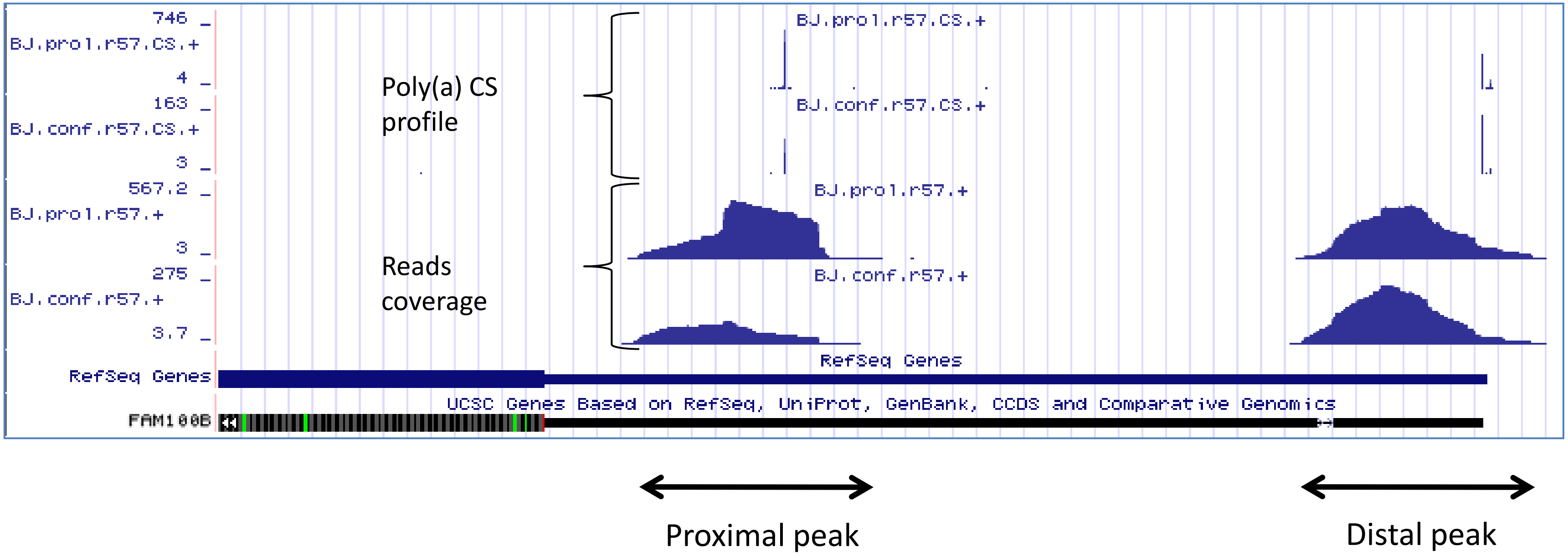

F

**FAM100B**	**Proximal poly(A) site**	**Distal poly(A) site**
**BJ proliferation**	1830	1811
**BJ confluent**	350	845

p= 1.88E-36

Figure S1

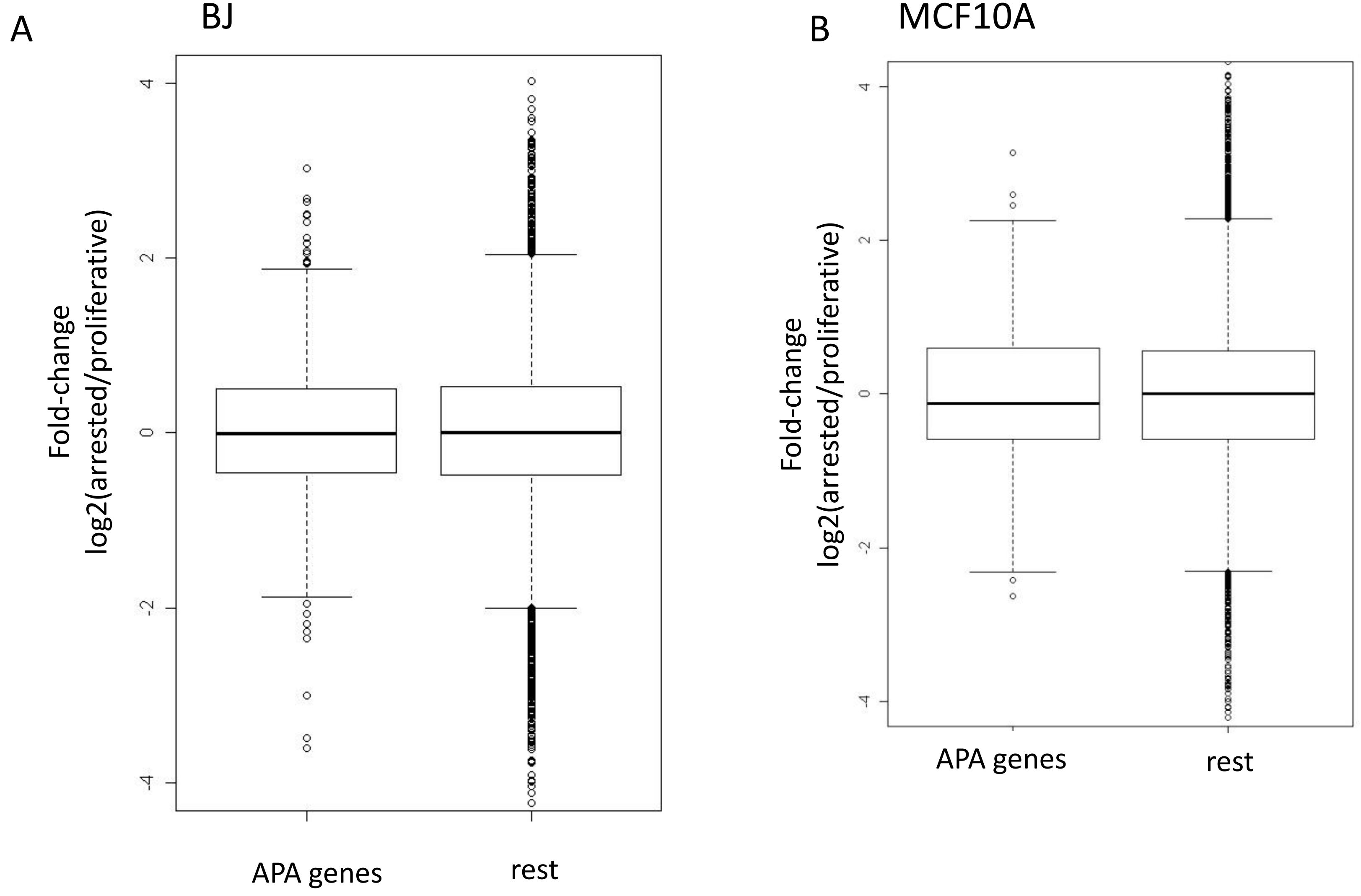
A
BJ
Fold-change
log2(arrested/proliferative)
4
2
0
-2
-4
APA genes
rest
B
MCF10A
Fold-change
log2(arrested/proliferative)
4
2
0
-2
-4
APA genes
rest

Figure S2

A.

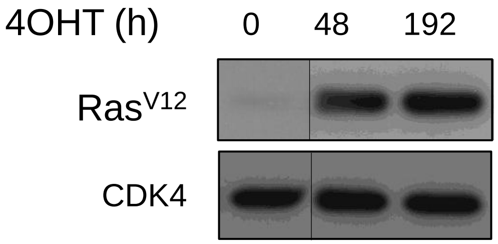

4OHT (h)
0
48
192
Ras^{V12}
CDK4

B.

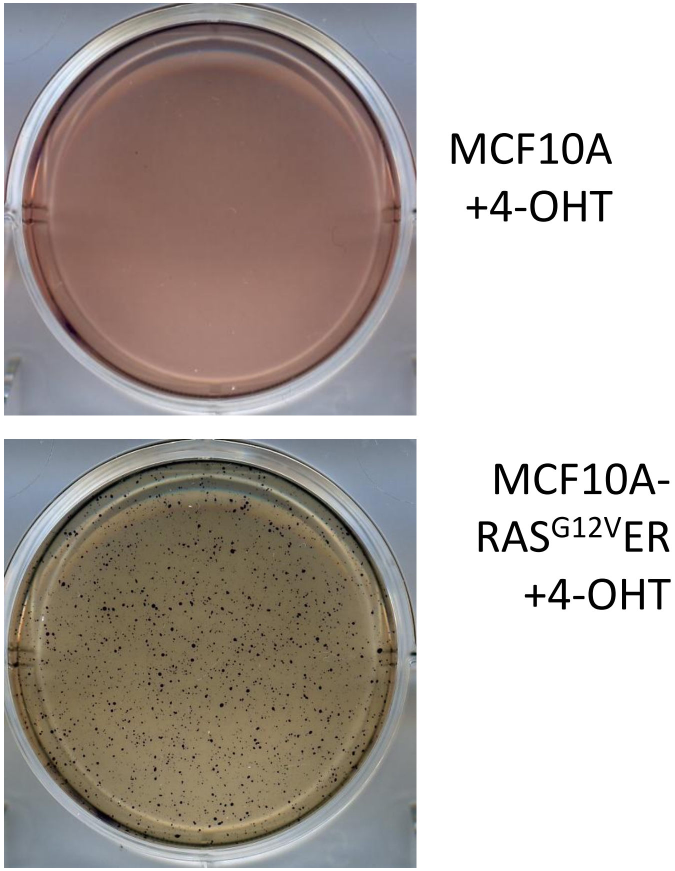

MCF10A
+4-OHT
MCF10A-
$RAS^{G12V}ER$
+4-OHT

Figure S3

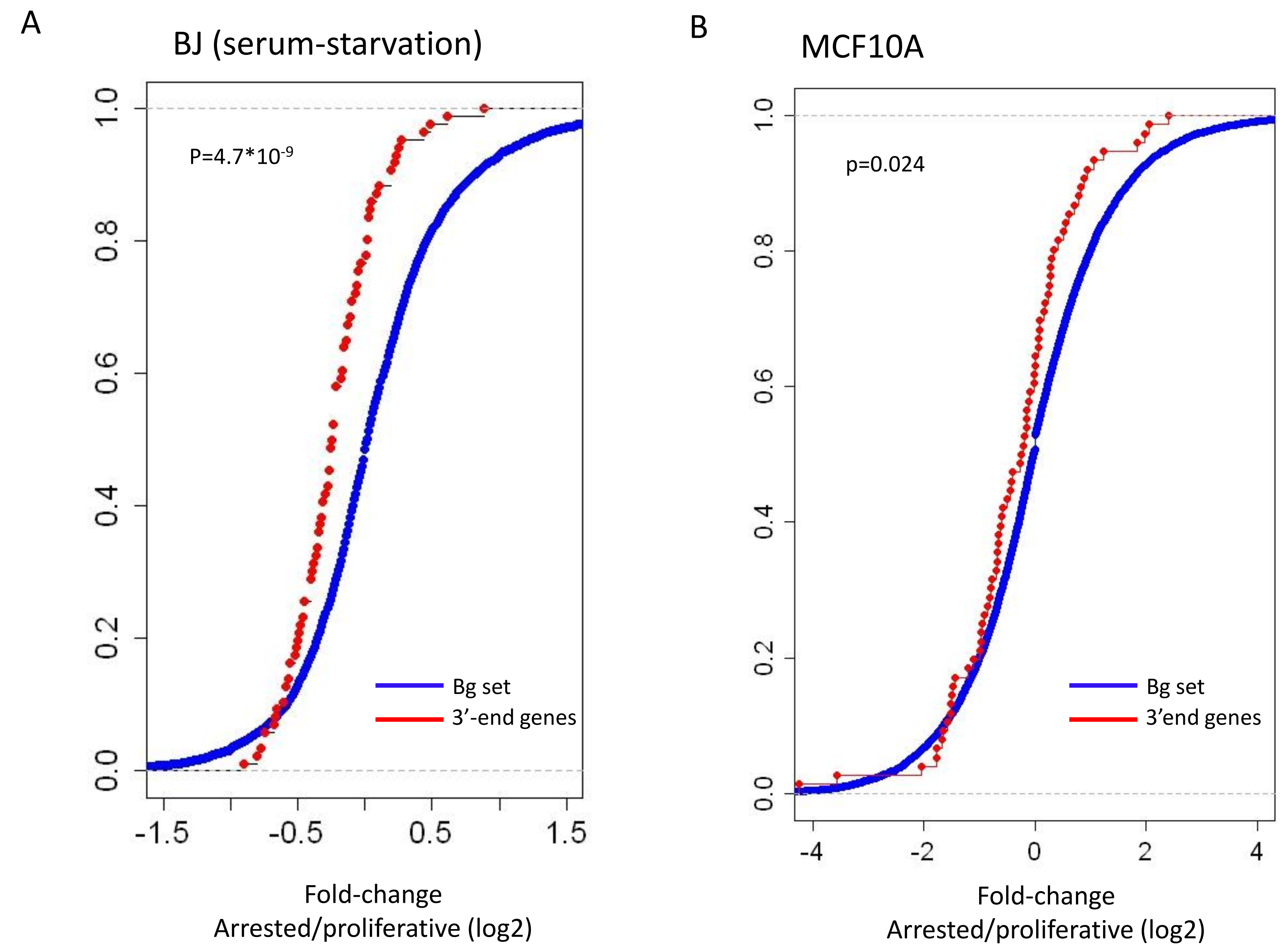
A
BJ (serum-starvation)
P=4.7*10-9
Bg set
3'-end genes
1.0
0.8
0.6
0.4
0.2
0.0
-1.5
-0.5
0.5
1.5
Fold-change
Arrested/proliferative (log2)
B
MCF10A
p=0.024
Bg set
3'end genes
1.0
0.8
0.6
0.4
0.2
0.0
-4
-2
0
2
4
Fold-change
Arrested/proliferative (log2)

Figure S4

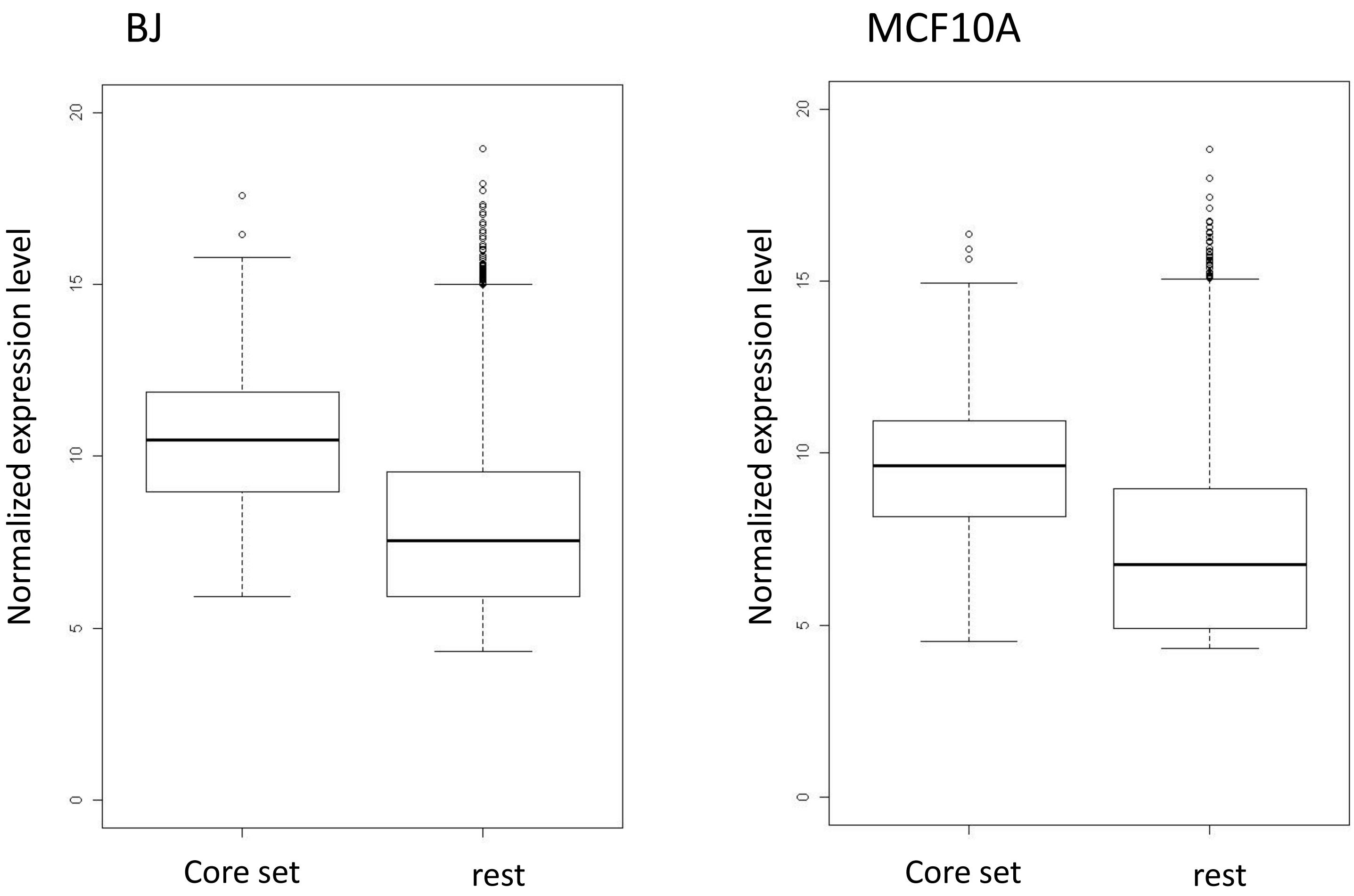
BJ
MCF10A
Normalized expression level
20
15
10
5
0
Core set
rest

Figure S5

Supplemental Figure Legends

Figure S1. A. Schematic description of the 3'-Seq method. **B.** An example for reads which span a poly(A) site and therefore allow its mapping with a nucleotide resolution. The canonical PAS upstream the cleavage site is indicated as well. The transcript's reference sequence is colored in blue below the aligned sequenced reads. **C.** An example for reads which stem from internal priming of the poly-dT primers to A-rich region within a transcript. Such reads were filtered out to avoid false calls of poly(A) sites. **D.** Enriched motifs identified on the set of CSs which mapped to introns, and their location distribution with respect to the mapped sites. **E.** An example for read coverage and poly(A) cleavage site profiles. Two poly(A) CSs were identified for the transcript shown here (FAM100B). **F.** Testing for shifts in usage of 3'UTR poly(A) sites between the examined conditions was done using chi-square test. The example shows the decrease in the usage of FAM100B proximal poly(A) site relative to the usage of the distal one in the transition of BJ cells from proliferative to confluent state.

Figure S2. No significant effect on gene expression levels was detected for the 3'UTR lengthening associated with proliferation arrest in either BJ (left) or MCF10A (right) cells. In both cases, fold-change distribution calculated for the set of transcripts that underwent 3'UTR lengthening did not significantly differ from the distribution calculated for the background set containing all the other transcripts in the dataset.

Figure S3. RAS^{G12V} induction and cellular transformation. A. MCF10A-RAS^{G12V}-ER cells were cultured in the presence or absence of 4-OHT for the indicated time periods, harvested and Immunoblot analysis was performed to detect RAS-ER and the control CDK4. **B.** MCF-10A and MCF10A-RAS^{G12V}-ER cells were plated in soft agar plates containing 4-OHT. Pictures were taken after 2 weeks.

Figure S4. Down-regulation of 3'-end processing genes upon serum starvation in BJ (A) and MCF10A (B) cells. This figure is the same as Figure 6A, but here calculation was done for the transition of BJ and MCF10A cells from proliferative to arrested state, induced by serum depletion. Here, for BJ cells (proliferating and serum-starved), gene expression levels were measured using rna-seq.

Figure S5. The statistical test applied for detection of shifts in poly(A) site usage is inherently less sensitive to changes in lowly expressed transcripts. Accordingly, the detected events were biased towards highly expressed genes.

Table S1. Summary of numbers of sequenced and uniquely mapped reads in the 3'-Seq experiments

	Reads length	Number of reads	Number of mapped reads	Number of uniquely mapped reads
**BJ proliferation**	35	27,416,302	24,064,349	10,730,927
**BJ confluent**	35	25,413,161	21,218,021	9,621,471
**BJ RAS-transformed**	35	26,165,194	22,670,994	11,180,489
**BJ proliferation**	100	75,157,030	41,705,981	29,582,142
**BJ confluent**	100	70,515,553	37,238,235	26,762,307
**MCF10A proliferation**	98	70,248,024	36,492,416	24,247,097
**MCF10A serum-starved**	98	49,911,326	20,170,825	13,592,273
**MCF10A RAS-transformed (0 days)**	98	64,782,691	28,640,391	18,560,756
**MCF10A RAS-transformed (2 days)**	98	55,089,264	25,921,230	16,952,246
**MCF10A RAS-transformed (8 days)**	98	65,208,661	27,490,250	20,911,698

Table S2

A. BJ cells

Enriched GO categories among genes down-regulated in the transition of BJ cells from proliferation to confluent state.

GO Term	Description	P-value
GO:0007049	cell cycle	3.27E-51
GO:0000279	M phase	3.36E-33
GO:0006260	DNA replication	3.57E-18
GO:0051726	regulation of cell cycle	6.73E-17
GO:0000075	cell cycle checkpoint	3.10E-14
GO:0006281	DNA repair	3.93E-12
GO:0006310	DNA recombination	5.08E-12
GO:0031055	chromatin remodeling at centromere	1.87E-11
GO:0034728	nucleosome organization	1.59E-10

Enriched GO categories among genes up-regulated in the transition of BJ cells from proliferation to confluent state.

GO Term	Description	P-value
GO:0007166	cell surface receptor linked signaling pathway	1.48E-08
GO:0032502	developmental process	2.90E-08
GO:0006695	cholesterol biosynthetic process	1.14E-07
GO:0009653	anatomical structure morphogenesis	1.26E-07
GO:0007155	cell adhesion	5.46E-07

B. MCF10A cells

Enriched GO categories among genes down-regulated in the transition of MCF10A cells from proliferation to serum-starved state.

GO Term	Description	P-value
GO:0007049	cell cycle	1.63E-18
GO:0007067	mitosis	3.86E-14
GO:0006260	DNA replication	5.41E-10
GO:0000075	cell cycle checkpoint	9.42E-08
GO:0009058	biosynthetic process	1.24E-07
GO:0000082	G1/S transition of mitotic cell cycle	2.96E-07
GO:0006364	rRNA processing	5.80E-07

Enriched GO categories among genes up-regulated in the transition of MCF10A cells from proliferation to serum-starved state.

GO Term	Description	P-value
GO:0007155	cell adhesion	3.52E-08
GO:0032502	developmental process	1.70E-07
GO:0033275	actin-myosin filament sliding	2.28E-07
GO:0048856	anatomical structure development	4.34E-07
GO:0006695	cholesterol biosynthetic process	1.08E-06
GO:0009888	tissue development	1.52E-06
GO:0030334	regulation of cell migration	3.33E-06
